# Anlotinib combined with transarterial chemoembolization for unresectable hepatocellular carcinoma associated with hepatitis B virus: a retrospective controlled study

**DOI:** 10.3389/fonc.2023.1235786

**Published:** 2023-11-22

**Authors:** Song Chen, Hongjie Cai, Zhiqiang Wu, Shuangyan Tang, Ludan Chen, Fan Wang, Wenquan Zhuang, Wenbo Guo

**Affiliations:** ^1^ Department of Minimally Invasive Interventional Therapy, State Key Laboratory of Oncology in South China, Guangdong Provincial Clinical Research Center for Cancer, Sun Yat-sen University Cancer Center, Guangzhou, China; ^2^ Department of Interventional Radiology, The First Affiliated Hospital of Sun Yat-sen University, Guangzhou, China

**Keywords:** anlotinib, transarterial chemoembolization, unresectable hepatocellular carcinoma, combination therapy, hepatitis B virus

## Abstract

**Purpose:**

To investigate the efficacy and safety of combined treatment of anlotinib and transarterial chemoembolization (TACE) in patients with unresectable hepatocellular carcinoma (uHCC) associated with hepatitis B virus (HBV) infection.

**Methods:**

We retrospectively collected the data of 96 uHCC patients associated with HBV infection who received either TACE only (TO group; n = 64) or anlotinib combined with TACE (TA group; n = 32) from January 2017 to January 2021. The primary endpoint was overall survival (OS). The secondary outcomes included progression-free survival (PFS), tumor response according to modified Response Evaluation Criteria in Solid Tumors (mRECIST) and RECIST 1.1, and adverse events (AEs).

**Results:**

The median OS and median PFS were significantly longer in the TA group compared to the TO group (17.6 months *vs.* 9.4 months, *p* = 0.018; 6.7 months *vs.* 3.8 months, *p* = 0.003, respectively). In addition, the overall objective response rate (ORR) and disease control rate (DCR) numerically increased in the TA group (mRECIST, ORR 65.6% *vs.* 46.9%, *p* = 0.064, DCR 90.6% *vs.* 85.9%, *p* = 0.382; RECIST 1.1, ORR 46.9% *vs.* 15.6%, *p* = 0.001, DCR 90.6% *vs.* 85.9%, *p* = 0.382, respectively). It was worth noting that no treatment-related mortality occurred during the study. The most common AEs included elevated transaminases (56.3%), decreased appetite (46.9%), and abdominal pain (37.5%) in the TA group. Although the incidence rate of grade 3/4 AEs was higher in the TA group, all of them were controllable.

**Conclusions:**

The combination of anlotinib and TACE has shown promising results in improving outcomes for patients with HBV-related uHCC, as compared to TACE monotherapy. In addition, this combination therapy has demonstrated a controllable safety profile. However, further validation is urgently needed through randomized controlled trials with large sample sizes.

## Graphical Abstract

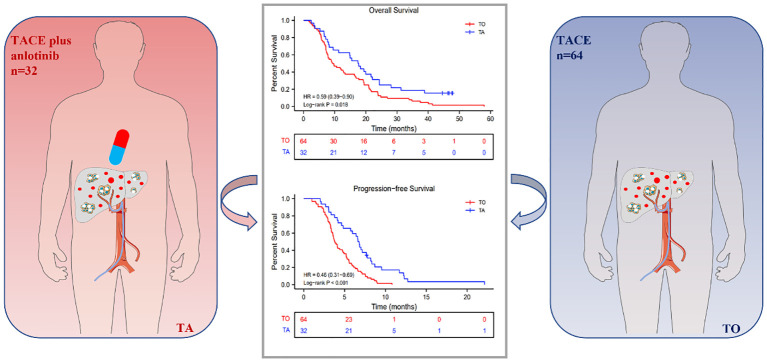

## Introduction

Hepatocellular carcinoma (HCC) is a highly prevalent and deadly form of cancer, ranking sixth and third in terms of incidence and mortality, respectively, among all malignant tumors worldwide. China has the ninth-highest incidence of HCC globally and has over 50% of new HCC cases reported worldwide each year (1). Generally, conventional curative treatment options for HCC included ablation, resection, and transplantation, although the majority of patients are ineligible due to various factors, including tumor size, location, number, liver function, extrahepatic metastases, vascular involvement, and overall patient condition (2, 3).

The Barcelona Clinic Liver Cancer (BCLC) staging system is widely recognized and utilized in clinical practice, and it is also commonly employed in clinical trials for the treatment of HCC (4, 5). According to the BCLC staging system, transarterial chemoembolization (TACE) is recommended as the standard option for intermediate-stage (BCLC-B stage) HCC, while it has been extended for patients with almost all unresectable HCC (uHCC) in many countries, with numerous clinical studies reporting survival advantages in comparison to conservative management or other regimens (6–9). However, based on previously routine TACE, the majority of patients experienced a rapid relapse and poor prognosis within a relatively short period, resulting in a push for the exploration of other feasible options (10, 11).

To our knowledge, TACE exerts therapeutic effect mainly based on constructing intratumoral hypoxia and ischemia environment, which could induce the upregulation of vascular endothelial growth factor (VEGF) and fibroblast growth factor (FGF) at the same time, further promoting tumor growth, invasion, and metastasis. Tyrosine kinase inhibitors (TKIs) can effectively decrease VEGF and FGF, so the combination with TACE has a synergistic antitumor effect theoretically. Although several prospective clinical trials have been reported with negative results regarding the superiority of combination treatment compared to TACE alone, especially for sorafenib, more and more clinical trials presented compelling clinical evidence indicating that patients with uHCC can benefit more from the combination of TACE and TKIs when compared to TACE monotherapy (12–16).

Anlotinib is a novel small-molecule TKI that selectively targets vascular endothelial growth factor receptors (VEGFRs), fibroblast growth factor receptors (FGFRs), platelet-derived growth factor receptors (PDGFRs), and c-kit receptors, demonstrating promising efficacy in treating a variety of malignancies, including advanced non-small cell lung cancer (NSCLC), medullary thyroid carcinoma, soft tissue sarcoma (STS), metastatic cervical cancer, neuroblastoma, and advanced biliary tract cancers (17–19). Recently, anlotinib has shown its efficacy and safety for patients with uHCC as well, especially in combination with TACE (20, 21).

Although vaccination programs have been implemented and new infections among children have decreased obviously, the percentage of people living with chronic hepatitis B virus (HBV) infection worldwide remained as high as 3.5% of the global population in 2015 (22). Persistent HBV infection is actually responsible for over 50% of all HCC cases worldwide and up to 85% in some areas where the infection is endemic, such as in China (23). Therefore, HCC associated with HBV infection is the major burden for HCC management in China, and the choice of treatment regimens ranks as extremely important.

Anlotinib combined with TACE may have a superior synergistic antitumor effect, but no clinical study has reported the long-term survival of the combination therapy yet. Therefore, our study aims to investigate the efficacy and safety of the combination therapy compared with TACE monotherapy in patients with uHCC associated with HBV infection.

## Patients and methods

### Patient eligibility

We retrospectively collected uHCC patients who underwent either TACE only or a combination of anlotinib and TACE from January 2017 to January 2021 at the First Affiliated Hospital of Sun Yat-sen University. The unresectable criteria include one or more of the following aspects: i) residual liver volume is insufficient, ii) distant metastasis or macrovascular invasion, iii) liver function or physical condition is poor, and iv) resection is highly risky as assessed by two experienced surgeons.

All patients were preoperatively evaluated by MRI, abdominal dynamic CT, and/or abdominal ultrasonography. The criteria for inclusion were as follows: 1) pathologically or radiologically diagnosed with intermediate to advanced HCC consistent with the European Association for the Study of the Liver (EASL), 2) deemed to be unresectable or incurable according to the above description, 3) liver function scored as Child-Pugh class A or B, 4) performance status score of Eastern Cooperative Oncology Group (ECOG PS) of 0 or 1, and 5) infected with HBV. Exclusion criteria were as follows: 1) <18 years old or ≥75 years old, 2) previous antitumor therapy of any kind, 3) contraindicated to receive TACE or anlotinib, 4) anlotinib administration less than 4 weeks, 5) discontinued anlotinib due to personal reason, 6) incomplete follow-up medical data, and 7) malignant tumors in other organs.

The study was approved by the ethics committee at the First Affiliated Hospital of Sun Yat-sen University, and all recruited patients provided informed consent.

### Treatment protocol

#### TACE therapy

Each patient enrolled underwent at least one TACE session. The catheter tip was selectively or superselectively inserted into the tumor-feeding artery branches based on tumor location, size, and blood supply. The chemoembolization regimen was first performed by emulsion consisting of pharmorubicin and lipiodol and subsequently embolizing the trunk with a microsphere or absorbable gelatin sponge. The embolization endpoint was classified according to the previously established subjective angiographic chemoembolization endpoint (SACE) scale. Generally, the embolization endpoint reached SACE level III or IV, denoting reduced or none antegrade arterial flow without tumor blush (24). All procedures were operated by a physician. Efficacy assessment was performed every 4–6 weeks after TACE, and patients received on-demand TACE according to the investigator’s assessment.

#### Anlotinib therapy

Patients in the TA group were informed about the economic cost, expected outcomes, and possible side effects of anlotinib. With the patients’ consent, anlotinib was initially administered (12 mg) once a day for 3 to 5 days after the first TACE session (2 weeks on and 1 week off). If mild to moderate adverse events (AEs) of grade 1/2 occurred, the frequency and dose of anlotinib would be the same as before, but the side effects were promptly addressed. In the event of severe AEs of grade 3/4, the dose of anlotinib would be decreased to 8 mg once a day, or the frequency would be reduced to every 2 days until the AEs were resolved or relieved. If the symptoms persisted, the administration of anlotinib would be temporarily halted until the AEs were alleviated or resolved.

#### Antiviral therapy

Because all patients enrolled in the study were infected with HBV, they received routine antiviral medication therapy (entecavir or tenofovir alafenamide fumarate) every day.

### Assessment and follow-up

The follow-ups were performed every 4–6 weeks after each TACE session, while the interval for the next follow-ups was extended to every 9–12 weeks when patients achieved stable disease. Imaging examination including abdominal MRI with contrast, dynamic CT scans of the chest and abdomen, and/or abdominal ultrasound was used to evaluate the progression-free survival (PFS) and tumor response based on modified Response Evaluation Criteria in Solid Tumors (mRECIST) and RECIST 1.1 criteria. Serum tests were also performed to assess the effectiveness and safety, including liver function, alpha-fetoprotein (AFP) levels, and blood cell count.

Objective response rate (ORR) was defined as the combined percentage of partial response (PR) and complete response (CR). Disease control rate (DCR) was defined as the combined percentage of stable disease (SD) and ORR. Overall survival (OS) refers to the length of time from the start of treatment until death for any reason. PFS refers to the length of time from the start of treatment until either tumor progression or death for any reason. AEs were assessed based on their frequency and severity grade using the Common Terminology Criteria for Adverse Events (CTCAE; version 5.0).

### Statistical analysis

R statistical software (version 4.0.3; R Foundation Inc., Vienna, Austria) and SPSS version 25.0 software (SPSS, Chicago, IL, USA) for the statistical analysis were used. The median values and interquartile ranges for the clinical parameters were computed, and then a Student’s t-test was performed to compare the continuous variables between the two groups. Survival analyses, including OS and PFS, were performed using the Kaplan–Meier method, and differences were analyzed by the log-rank test. Cox regression analyses were conducted to identify factors associated with survival outcomes. Factors with a *p*-value < 0.05 in univariate analysis were included in multivariate analysis. The therapeutic efficacy was demonstrated by the ORR and DCR, which were compared using the chi-square test. Statistical significance was defined as two-tailed *p* < 0.05.

## Results

### Patient characteristics

**Figure 1:**
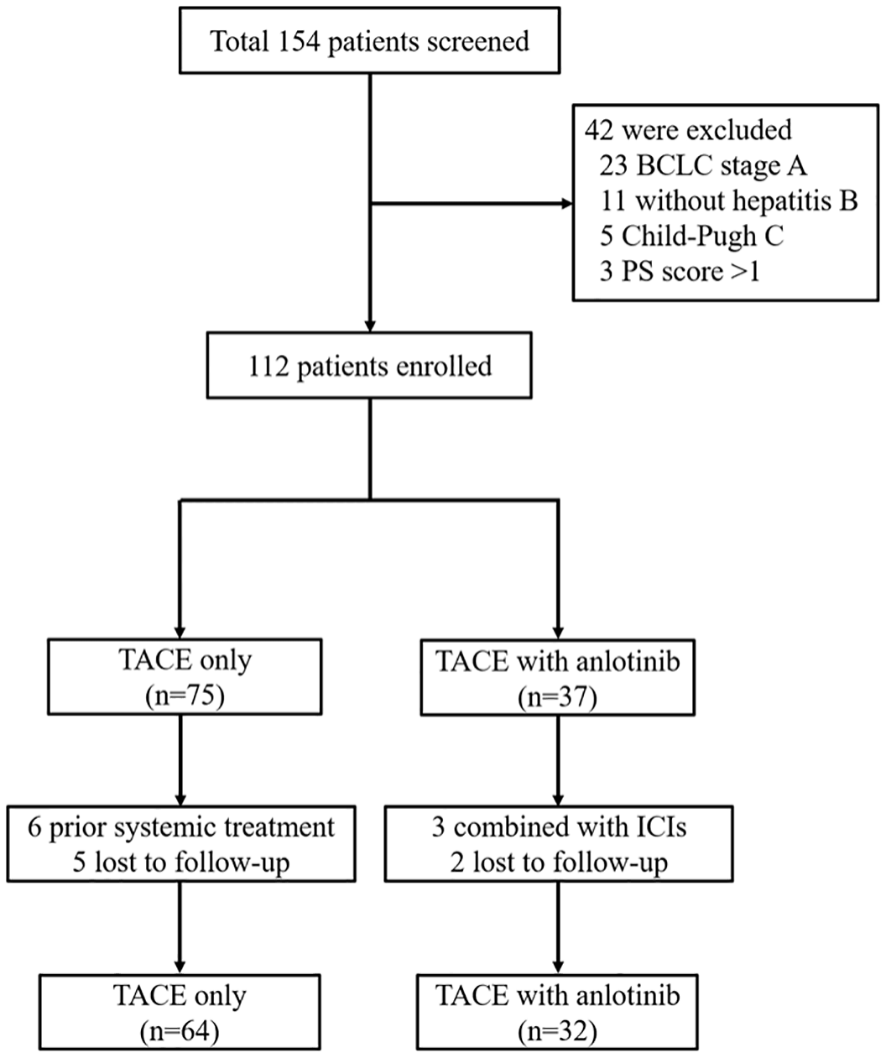
Patient enrollment flow diagram. TACE, transarterial chemoembolization; BCLC, Barcelona Clinic Liver Cancer; PS, performance status; ICIs, immune checkpoint inhibitors.

From January 2017 to January 2021, a total of 154 patients with unresectable treated with TO or TA were screened. Among them, 96 patients were finally eligible and enrolled in the current study, with 64 receiving TACE monotherapy (TO group) and the other 32 patients receiving combination therapy (TA group) (**Figure 1**).

The baseline characteristics of all patients in the two groups are listed in **Table 1**. All of the baseline characteristics were well balanced between the two groups including age, gender, BCLC stage, Child-Pugh class, albumin–bilirubin (ALBI) grade, PS score, tumor number, largest size, AFP level, portal vein tumor thrombus (PVTT), and extrahepatic spread. Obviously, the tumor burden was relatively high in the two groups with the median largest size over 11 cm.

**Table 1:** Baseline characteristics of patients in TO and TA groups.

Characteristic	TO (n = 64)	TA (n = 32)	*p*
Age, mean ± SD	53.4 ± 12.4	53.6 ± 13.6	0.960
Gender, n (%)			0.172
Female	5 (7.8%)	6 (18.8%)	
Male	59 (92.2%)	26 (81.2%)	
BCLC stage, n (%)			1.000
B	32 (50.0%)	16 (50.0%)	
C	32 (50.0%)	16 (50.0%)	
CNLC stage, n (%)			0.912
IIb	32 (50.0%)	16 (50.0%)	
IIIa	24 (37.5%)	11 (34.4%)	
IIIb	8 (12.5%)	5 (15.6%)	
Child-Pugh, n (%)			0.636
A	54 (84.4%)	25 (78.1%)	
B	10 (15.6%)	7 (21.9%)	
ALBI grade, n (%)			0.206
1	6 (9.4%)	6 (18.8%)	
2	58 (90.6%)	26 (81.2%)	
Performance status, n (%)			0.527
0	57 (89.1%)	27 (84.4%)	
1	7 (10.9%)	5 (15.6%)	
Tumor number, n (%)			1.000
Multiple	55 (85.9%)	28 (87.5%)	
Solitary	9 (14.1%)	4 (12.5%)	
Largest size (cm), median (Q1, Q3)	11.5 (8.3, 14.3)	11.5 (8.7, 13.1)	0.756
AFP, n (%)			1.000
<400	25 (39.1%)	13 (40.6%)	
≥400	39 (60.9%)	19 (59.4%)	
PVTT, n (%)			0.827
Absence	35 (54.7%)	19 (59.4%)	
Presence	29 (45.3%)	13 (40.6%)	
Extrahepatic spread, n (%)			0.755
No	56 (87.5%)	27 (84.4%)	
Yes	8 (12.5%)	5 (15.6%)	

TO, TACE only; TA, TACE combined with anlotinib; TACE, transarterial chemoembolization; BCLC, Barcelona Clinic Liver Cancer; CNLC, China Liver Cancer Staging; ALBI, albumin–bilirubin; AFP, alpha-fetoprotein; PVTT, portal vein tumor thrombus.

### Efficacy outcomes

The last follow-up date was September 30, 2022. The median follow-up durations in the TO and TA groups were 24.0 months (range, 8.0 to 30.5 months) and 17.5 months (range, 5.0 to 25.4 months), respectively. The median anlotinib treatment duration was 10.7 months (range, 3.8 to 21.9 months).

**Figure 2:**
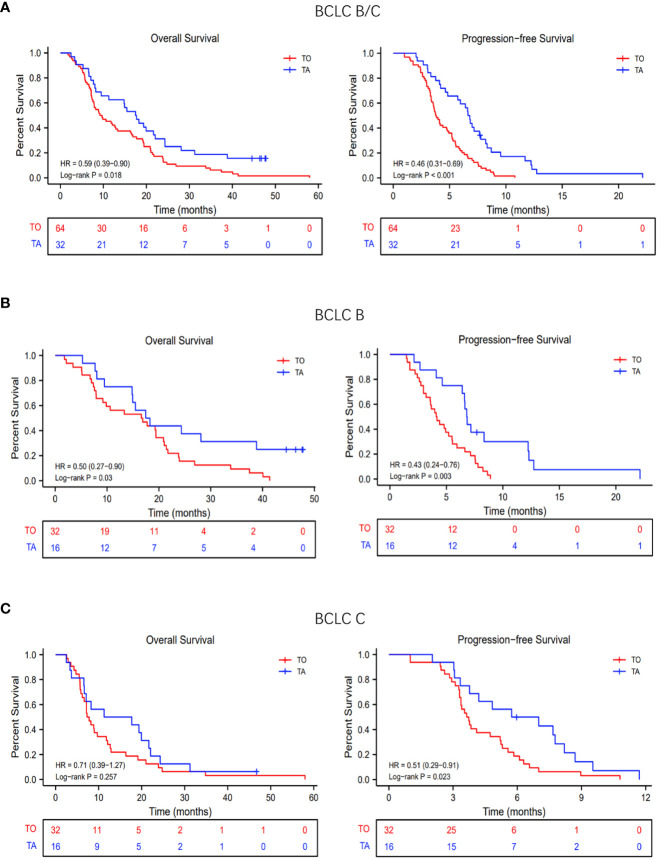
Overall survival (OS) and progression-free survival (PFS) with TO or TA regimen. **(A)** The OS and PFS of all patients. **(B)** The OS and PFS of patients with BCLC stage **(B, C)** The OS and PFS of patients with BCLC stage **(C)** TACE, transarterial chemoembolization; TO, TACE only; TA, TACE combined with anlotinib; BCLC, Barcelona Clinic Liver Cancer.

**Figure 3:**
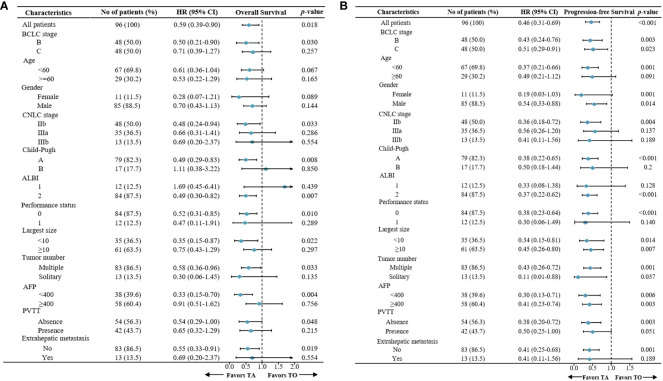
Forest plot for **(A)** overall survival and **(B)** progression-free survival of the patients treated with TA or TO. TACE, transarterial chemoembolization; TO, TACE only; TA, TACE combined with anlotinib.

In the TA and TO groups, the median OS was 9.4 months (95% confidence interval [CI], 7.8 to 16.3 months) and 17.6 months (95% CI, 11.3 to 24.3 months), respectively. Thus, OS was significantly prolonged in the TA group (hazard ratio [HR]: 0.50, 95% CI: 0.27–0.90; *p* = 0.03; **Figure 2A**). The median PFS was also longer (HR: 0.43, 95% CI: 0.24–0.76; *p* = 0.003; **Figure 2A**) in the TA group (3.8 months, 95% CI: 3.5 to 4.9 months) compared with the TO group (6.7 months, 95% CI: 57 to 8.2 months). In the subgroup analysis, based on the BCLC stage (BCLC B/C), the OS and PFS were also longer in the TA group, consistent with the overall population (**Figures 2B, C**). The forest plot analysis of factors associated with OS and PFS is exhibited in **Figure 3**. TA provided a clinical benefit in patients with all characteristics except for OS in Child-Pugh class B and ALBI grade 1.

All the patients were evaluated for tumor response in the two groups. According to mRECIST, the ORR and DCR were 65.6% *vs.* 46.9% (*p* = 0.064) and 90.6% *vs.* 85.9% (*p* = 0.382), respectively. According to RECIST 1.1, the ORR and DCR were 46.9% *vs.* 15.6% (*p* = 0.001) and 90.6% *vs.* 85.9% (*p* = 0.382), respectively (**Table 2**). In the subgroup analysis, the ORR and DCR were higher in the TA group whether for the BCLC-B stage or BCLC-C stage (**Table 2**).

**Table 2:** Tumor response in the total and subgroups according to mRECIST and RECIST 1.1.

	mRECIST									RECIST								
	Total			BCLC B			BCLC C			Total			BCLC B			BCLC C		
	TO (n = 64)	TA (n = 32)	*p*	TO (n = 32)	TA (n = 16)	*p*	TO (n = 32)	TA (n = 16)	*p*	TO (n = 64)	TA (n = 32)	*p*	TO (n = 32)	TA (n = 16)	*p*	TO (n = 32)	TA (n = 16)	*p*
CR	0 (0)	3 (9.4%)		0 (0)	1 (6.3%)		0 (0)	2 (12.5%)		0 (0)	0 (0)		0 (0)	0 (0)		0 (0)	0 (0)	
PR	30 (46.9%)	18 (56.2%)		17 (53.1%)	12 (75.0%)		13 (40.6%)	6 (37.5%)		10 (15.6%)	15 (46.9%)		6 (18.8%)	9 (56.3%)		4 (12.5%)	6 (37.5%)	
SD	25 (39.0%)	8 (25.0%)		11 (34.4%)	2 (12.4%)		14 (43.8%)	6 (37.5%)		45 (70.3%)	14 (43.7%)		22 (68.7%)	6 (37.5%)		23 (71.9%)	8 (50.0%)	
PD	9 (14.1%)	3 (9.4%)		4 (12.5%)	1 (6.3%)		5 (15.6%)	2 (12.5%)		9 (14.1%)	3 (9.4%)		4 (12.5%)	1 (6.2%)		5 (15.6%)	2 (125%)	
ORR	30 (46.9%)	21 (65.6%)	0.064	17 (53.1%)	13 (81.3%)	0.055	13 (40.6%)	8 (50.0%)	0.378	10 (15.6%)	15 (46.9%)	0.001	6 (18,8%)	9 (56.3%)	0.011	4 (12.5%)	6 (37.5%)	0.054
DCR	55 (85.9%)	29 (90.6%)	0.382	28 (87.5%)	15 (93.8%)	0.454	27 (84.4%)	14 (87.5%)	0.571	55 (85.9%)	29 (90.6%)	0.382	28 (87.5%)	15 (93.8%)	0.454	27 (84.4%)	14 (87.5%)	0.571

mRECIST, modified Response Evaluation Criteria in Solid Tumors; BCLC, Barcelona Clinic Liver Cancer; TACE, transarterial chemoembolization; TO, TACE only; TA, TACE combined with anlotinib; CR, complete response; PR, partial response; SD, stable disease; PD, progressive disease; ORR, objective response rate; DCR, disease control rate.

### Univariate and multivariate analyses for survival

**Supplementary Table 1** demonstrates the univariate and multivariate analyses for survival. Multivariate analysis demonstrated that the therapeutic regimen was an independent risk factor for both OS (HR 0.58, 95% CI, 0.37–0.91; *p* = 0.018) and PFS (HR 0.39, 95% CI, 0.24–0.62; *p* < 0.001). Multivariate analysis showed that AFP ≥ 400 was a risk factor for PFS (HR 1.80, 95% CI, 1.16–2.78; *p* = 0.008).

### Progression reason analysis

As for the progression reason analysis, there were a total of four ways to progress: local lesion progression, intrahepatic metastasis, extrahepatic metastasis, and death. In the two groups, the proportion was 31.3%, 46.9%, 12.5%, and 9.3%, respectively, *vs.* 25.0%, 31.3%, 12.4%, and 31.3%, respectively (*p* = 0.054). Obviously, the proportion of local lesion progression and intrahepatic metastasis in the TA group was less than that in the TO group (**Supplementary Figure 1**; **Table 3**).

**Table 3:** Different endpoints to progression-free survival.

	TO (n = 64)	TA (n = 32)	*p*
Local lesion progression	20 (31.3%)	8 (25.0%)	
Intrahepatic metastasis	30 (46.9%)	10 (31.3%)	
Extrahepatic metastasis	8 (12.5%)	4 (12.4%)	
death	6 (9.3%)	10 (31.3%)	0.054

Data are presented as n (%),

TO, TACE only; TA, TACE combined with anlotinib; TACE, transarterial chemoembolization.

### Subsequent treatment

After tumor progression, 55 patients (85.9%) in the TO group and 30 patients (93.8%) in the TA group received subsequent treatment. With the positive results of the REFLECT study (25), a considerable proportion of the patients converted to lenvatinib with or without programmed death protein 1 (PD-1) after progression. In the TO group, the most common subsequent therapy was anlotinib combined with TACE (**Supplementary Table 2**).

### Safety

All AEs were evaluated as mild to moderate and controllable, with no treatment-associated death occurring. Although more patients experienced AEs in the TA group, especially for grade 3/4 AEs, none of the patients discontinued therapy. The most common AEs included elevated transaminases (56.3%), decreased appetite (46.9%), and abdominal pain (37.5%) in the TA group. The details of AEs are summarized in **Table 4**.

**Table 4:** Treatment-related adverse events.

AEs	Any grade			Grade 3/4		
	TO (N = 64)	TA (N = 32)	*p*	TO (N = 64)	TA (N = 32)	*p*
Abdominal pain	25 (39.1)	12 (37.5)	0.882	3 (4.7)	1 (3.1)	<0.001
Nausea	22 (34.4)	10 (31.3)	0.759	0 (0)	0 (0)	1.000
Diarrhea	12 (18.8)	7 (21.9)	0.717	0 (0)	1 (3.1)	0.333
Decreased appetite	20 (31.3)	15 (46.9)	0.165	1 (1.6)	2 (6.3)	0.534
Erythra	0 (0)	5 (15.6)	0.006	0 (0)	2 (6.3)	0.109
Fatigue	6 (9.4)	5 (15.6)	0.571	0 (0)	0 (0)	1.000
Hypoproteinemia	12 (18.8)	5 (15.6)	0.656	2 (3.1)	0 (0)	0.551
Elevated bilirubin	10 (15.6)	6 (18.8)	0.699	1 (1.6)	1 (3.1)	1.000
Elevated transaminases	30 (46.9)	18 (56.3)	0.386	3 (4.7)	2 (6.3)	0.749
Hypothyroidism	0 (0)	6 (18.8)	0.001	0 (0)	1 (3.1)	0.333
Decreased PLT	9 (14.1)	8 (25.0)	0.186	0 (0)	0 (0)	1.000
Hypertension	3 (4.7)	6 (18.8)	0.063	0 (0)	0 (0)	1.000
Hand–foot skin reaction	0 (0)	8 (25.0)	<0.001	0 (0)	2 (6.3)	0.109
Dysphonia	0 (2)	2 (6.3)	0.109	0 (0)	0 (0)	1.000
Proteinuria	2 (3.1)	5 (15.6)	0.071	0 (0)	0 (0)	1.000
Bleeding (gingiva)	1 (1.6)	4 (12.5)	0.081	0 (0)	1 (3.1)	0.333
Joint pain	0 (4)	4 (12.5)	0.012	0 (0)	0 (0)	1.000

TO, TACE only; TA, TACE combined with anlotinib; TACE, transarterial chemoembolization; AEs, adverse events; PLT, platelet count.

## Discussion

We report here the primary results of a retrospective controlled study of anlotinib combined with TACE versus TACE monotherapy in patients with uHCC associated with HBV infection. Anlotinib combined with TACE demonstrated a significant improvement in OS, PFS, and tumor response, further confirming the benefits observed in our previous investigations (20). The study revealed that combination therapy has emerged as a significant independent prognostic factor for improved survival with a manageable safety profile. In summary, we found that anlotinib combined with TACE provided more clinical benefits than TACE monotherapy for patients with uHCC associated with HBV infection with acceptable AEs.

TACE is indeed recommended as the standard treatment for intermediate-stage HCC, while it is the most commonly served as a first-line treatment for uHCC in real-world clinical practice (26). However, TACE monotherapy remains to be further improved with unsatisfactory effectiveness in local recurrence and liver function damage, and TACE combined with TKIs has shown promising prognosis based on the synergistic antitumor effect in previous studies (12–16). The use of drug-eluting beads (DEB-TACE) might contribute to improved outcomes, while it has not been reported with superiority over conventional TACE (cTACE) in terms of survival yet (27, 28), so we opted for cTACE as the standard procedure in our study. There were several reasons for choosing anlotinib combined with TKIs. First, at the time when we decided to investigate the effectiveness of TACE plus TKIs, lenvatinib was not approved by the National Medical Products Administration (NMPA) because the results of the REFLECT study were not reported then. Second, we tried to combine TACE with sorafenib, but the majority of patients refused to continue due to serious side effects and high cost, while anlotinib was approximately one-tenth of the annual economic cost compared to sorafenib with fewer adverse events when first approved in China, which was highly cost-effective and more acceptable. Third, anlotinib is a novel multi-target TKI, which plays a crucial role in inhibiting elements implicated in tumor angiogenesis and signals promoting tumor proliferation, just as lenvatinib and sorafenib.

In both the overall population and the BCLC-B stage subpopulation, the median OS was significantly prolonged in the TA group (overall population, 17.6 *vs.* 9.4 months, *p* = 0.0183; BCLC-B subpopulation, 17.9 *vs.* 16.7 months, *p* = 0.03), while there was no significant difference in median OS between the TA and TO groups (14.5 *vs.* 7.5 months, *p* = 0.257) in the BCLC-C stage subpopulation. On the one hand, the investigated sample was relatively small, influencing the final result. On the other hand, TACE might not be the preferred option for advanced-stage patients, especially for those with vp3/4 portal vein tumor thrombus (PVTT) (29, 30). However, there was an obvious trend for prolonged survival, indicating the advantage of combination therapy. Furthermore, the LAUNCH study has demonstrated that TACE combined with TKIs contributed more for advanced patients compared to TKIs monotherapy (31).

In the other subgroup analysis, significant differences were not observed in certain subgroups with small proportional cohorts due to limitations in the number of cases. In general, anlotinib combined with TACE provided survival advantages in patients except for those with Child-Pugh class B or ALBI grade I liver function, indicating that we should take liver function into more consideration when choosing therapy regimens. Additionally, monotherapy might be a preferred option for patients with poor liver function, given that it causes minimal damage to liver function, consistent with a previous study (10, 32). Univariate and multivariate Cox regression analyses indicated that the treatment regimen has emerged as the only significant prognostic factor associated with both PFS and OS, which further confirmed the importance of combination therapy rather than other characteristics in improving efficacy.

In the analysis of different endpoints to PFS, it was obvious that the local lesion progression and intrahepatic metastasis just made up a small proportion in the TA group compared to the TO group. The finding suggested that anlotinib, as an anti-angiogenesis TKI, might have a greater ability to control the recurrence of local or intrahepatic lesions, indicating the synergistic antitumor effect combined with TACE. As reported, local and intrahepatic recurrences principally limited the survival benefit conferred by TACE (33), so the combination of anlotinib might improve prognosis effectively.

Additionally, with the successful prospective trials of anti-programmed death 1 (anti-PD-1) and anti-programmed death ligand 1 (anti-PD-L1) for advanced HCC, the treatment for HCC has stepped into the novel era of immunotherapy (34). Except for the combination of targeted therapy and immunotherapy, locoregional therapy combined with targeted therapy and immunotherapy has also been reported with many positive outcomes (35, 36). The CHANCE001 study demonstrated that TACE combined with PD-(L)1 and molecularly targeted agents (MTAs) significantly improves outcomes versus TACE monotherapy for Chinese patients with uHCC in real-world practice, with an acceptable safety profile (37). Based on the above results, triple combination therapy will be explored next.

The ratio of subsequent therapy was higher in the TA group (93.8% *vs.* 85.9%), and it was also higher than that in the combination group (sorafenib combined with TACE) of the TACTICS trial (58.8%) (38), demonstrating that subsequent therapy after progression could be performed more frequently in patients treated with anlotinib plus TACE. There were two possible explanations. First, anlotinib combined with TACE provided superior efficacy. Second, anlotinib proved to be less toxic compared to sorafenib, both of which resulted in better patient compliance.

Compared to TACE monotherapy, there was no doubt that more AEs were observed in the combination group. However, compared with TACE combined with sorafenib, these toxicities were mild to moderate and acceptable (39), and no patient discontinued therapy due to the safety profile.

There were several limitations in our study. First, anlotinib has not been approved for HCC yet, but anlotinib combined with penpulimab has been approved in China (40). With more and more clinical studies, anlotinib will be approved for HCC soon or later. Second, respective studies with relatively small samples resulted in various biases affecting survival outcomes. Further validation of these results by multicenter prospective randomized clinical trials is required.

In conclusion, our study first demonstrated that combination therapy with anlotinib and TACE showed significantly better efficacy in terms of long-term survival and tumor response for uHCC patients associated with HBV infection compared to TACE monotherapy, and prospective randomized controlled trials for validation are further needed.

## Data availability statement

The raw data supporting the conclusions of this article will be made available by the authors, without undue reservation.

## Ethics statement

The studies involving humans were approved by the Ethics Committee of the First Affiliated Hospital of Sun Yat-sen University. The studies were conducted in accordance with the local legislation and institutional requirements. The participants provided their written informed consent to participate in this study.

## Author contributions

Conception/design: SC, WZ, and WG. Provision of the study material: SC, HC, WZ, ZW, and WG. Collection of assembly of data: FW, ST, and LC. Data analysis and interpretation: SC and ZW. Manuscript writing or revision: SC and HC. Final manuscript approval: all authors have given their endorsement.
