# Supplementary material for: Anlotinib combined with transarterial chemoembolization for unresectable hepatocellular carcinoma associated with hepatitis B virus: a retrospective controlled study
Source: Front Oncol. 2023 Nov 22;13:1235786. doi: 10.3389/fonc.2023.1235786 (PMC10702964; doi:10.3389/fonc.2023.1235786)
Supplement: Supplementary file 1 [file DataSheet_1.docx]

| Variables |  | Overall Survival Progression-free Survival | | | | | | |  |
| --- | --- | --- | --- | --- | --- | --- | --- | --- | --- |
|  |  | Univariate  (*p*-value) | Multivariate  (*p*-value) | HR  (95% CI) |  | Univariate  (*p*-value) | Multivariate  (*p*-value) | HR  (95% CI) | |
| Treatment, (TA/TO) |  | 0.020 | 0.018 | 0.58 (0.37-0.91) |  | <0.001 | <0.001 | 0.39 (0.24-0.62) |  |
| Age (y), (</≥60) |  | 0.100 |  |  |  | 0.370 |  |  | |
| Gender, (Female/Male) |  | 0. 072 |  |  |  | 0.085 |  |  | |
| BCLC stage, (B/C) |  | 0.020 | 0.579 | 0.78 (0.33-1.85) |  | 0.172 |  |  | |
| CNLC stage |  |  |  |  |  |  |  |  | |
| IIIb |  | Reference |  |  |  | Reference |  |  | |
| IIIa |  | 0.830 |  |  |  | 0.776 |  |  | |
| IIb |  | 0.086 |  |  |  | 0.482 |  |  | |
| Child-Pugh, (A/B) |  | 0.245 |  |  |  | 0.894 |  |  | |
| ALBI, (1/2) |  | 0.248 |  |  |  | 0.130 |  |  | |
| Performance status, (0/1) |  | 0.326 |  |  |  | 0.669 |  |  | |
| Largest size (cm), (</≥10) |  | 0.993 |  |  |  | 0.564 |  |  | |
| Tumor number, (Solitary/>Multiple) |  | 0.078 |  |  |  | 0.655 |  |  | |
| AFP, (≥/<400) |  | 0.293 |  |  |  | 0.016 | 0.008 | 1.80 (1.16-2.78) | |
| PVTT, (Presence/Absence) |  | 0.015 | 0.480 | 1.37 (0.57-3.25) |  | 0.096 |  |  | |
| Extrahepatic metastasis, (Yes/No) |  | 0.225 |  |  |  | 0.750 |  |  | |

**Table S1** Univariate and multivariate analysis of risk factors for overall survival and progression-free survival

*TO* TACE only; *TA* TACE combined with anlotinib; *TACE* transarterial chemoembolization; *BCLC* Barcelona Clinic Liver Cancer; *CNLC* China Liver Cancer Staging; *ALBI* Albumin-Bilirubin; *AFP* alpha-fetoprotein; *PVTT* portal vein tumor thrombus.

**Table S2** Subsequent treatment

|  | TO (n=64) | TA (n=32) |
| --- | --- | --- |
| Received subsequent treatment | 55 (85.9%) | 30 (93.8%) |
| Lenvatinib | 12 (18.8%) | 8 (25.0%) |
| Lenvatinib + PD-1 | 8 (12.5%) | 3 (9.4%) |
| Regorafenib | 0 (0) | 2 (6.3%) |
| Anlotinib + PD-1 | 6 (9.4%) | 5 (15.6%) |
| HAIC + lenvatinib | 3 (4.7%) | 4 (12.5%) |
| HAIC + anlotinib | 4 (6.3%) | 2 (6.3%) |
| TACE + anlotinib | 15 (23.4%) | 0 (0) |
| TACE + anlotinib + PD-1 | 5 (7.8%) | 3 (9.4%) |
| HAIC + lenvatinib + PD-1 | 1 (1.6%) | 2 (6.3%) |
| Atezolizumab + bevacizumab | 1 (1.6%) | 1 (3.1%) |
| Best support care | 9 (29.7%) | 2 (6.2%) |

*TO* TACE only; *TA* TACE combined with anlotinib; *TACE* transarterial chemoembolization; *HAIC* hepatic arterial infusion chemotherapy; *PD -1* programmed death protein 1.

**
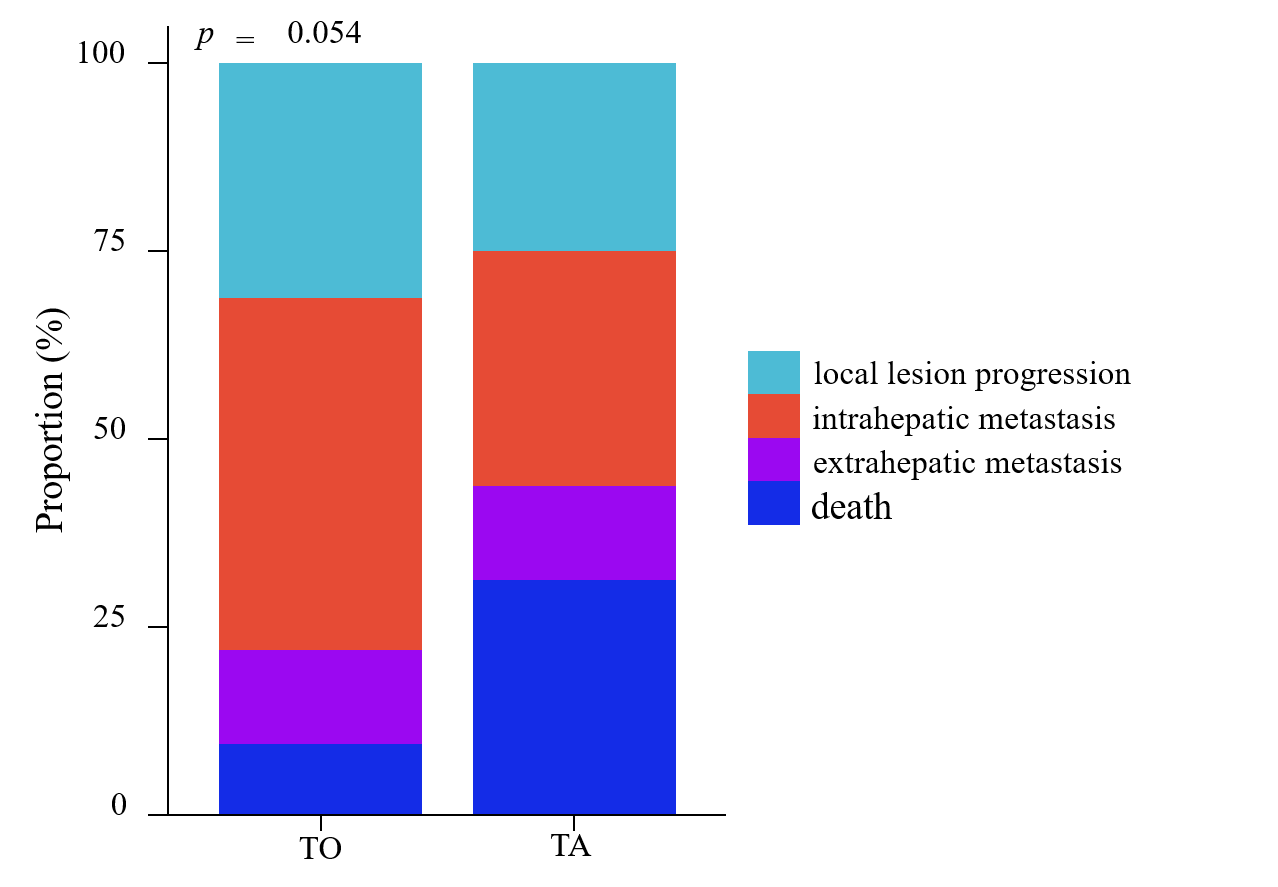
**

**Figure S1** The proportion of different endpoints to progression-free survival in TO and TA group. *TO* TACE only; *TA* TACE combined with anlotinib; *TACE* transarterial chemoembolization.
